# Characterization of 2-Path Product Signed Graphs with Its Properties

**DOI:** 10.1155/2017/1235715

**Published:** 2017-07-06

**Authors:** Deepa Sinha, Deepakshi Sharma

**Affiliations:** Department of Mathematics, South Asian University, Akbar Bhawan Chanakyapuri, New Delhi 110021, India

## Abstract

A* signed graph* is a simple graph where each edge receives a sign positive or negative. Such graphs are mainly used in social sciences where individuals represent vertices friendly relation between them as a positive edge and enmity as a negative edge. In signed graphs, we define these relationships (edges) as of friendship (“+” edge) or hostility (“−” edge). A* 2-path product signed graph *$S\hat{\#}S$ of a signed graph *S* is defined as follows: the vertex set is the same as *S* and two vertices are adjacent if and only if there exists a path of length two between them in *S*. The sign of an edge is the product of marks of vertices in *S* where the mark of vertex *u* in *S* is the product of signs of all edges incident to the vertex. In this paper, we give a characterization of 2-path product signed graphs. Also, some other properties such as sign-compatibility and canonically-sign-compatibility of 2-path product signed graphs are discussed along with isomorphism and switching equivalence of this signed graph with 2-path signed graph.

## 1. Introduction

Signed graph forms one of the most vibrant areas of research in graph theory and network analysis due to its link with behavioural and social sciences. The earliest appearance of signed graphs can be traced back to Heider [1] and Cartwright [2]. From that time to recently, signed theory has evolved rapidly with signed graphs being linked to algebra [3–5], social networks [6, 7], other models [8, 9], and graph spectra [10] to name few. In graph theory, itself signed graphs have been used to define many properties and new concepts. In [11, 12] the signed graph of line signed graphs is discussed, whereas [13, 14] talks about common edge signed graphs. The work in [15, 16] generalises the (*k*, *d*)-graceful graphs to signed graphs. The colouring of signed graphs is reported in [17–19]. The connection between the intersection graphs of neighborhood and signed graphs has also been studied [20–24]. Recently a Coxeter spectral analysis and a Coxeter spectral classification of the class of edge-bipartite graphs (that is a class of signed (multi)graphs) is developed in the papers [25–27] in relation to Lie theory problems, quasi Cartan matrices, Dynkin diagrams, Hilbert's X Problem, combinatorics of Coxeter groups, and the Auslander-Reiten theory of module categories and their derived categories. In this paper, we were mainly driven to carry out work in the area of signed graphs derived from 2-path product operations, which primarily deals with the structural reconfiguration of the structure of dynamical systems under prescribed rules and the rules are designed to address a variety of interconnections among the elements of the system. We have obtained some theoretical results (some of which are presented in [28]) with a hope of building necessary conceptual resources for applications. For standard terminology and notation in graph theory one can refer to Harary [29] and West [30] and for signed graph literature one can read Zaslavsky [19, 31, 32]. Throughout the text, we consider finite, undirected graph with no loops or multiple edges.

A* signed graph* is an ordered pair *S* = (Σ, *σ*), where Σ is a graph Σ = (*V*, *E*), called the underlying graph of *S* and *σ* : *E* → {+, −} is a function from the edge set *E* of Σ into the set {+, −}, called the* signature* (or *sign* in short) of *S*. Alternatively, the signed graph can be written as *S* = (*V*, *E*, *σ*), with *V*, *E*, and *σ* in the above sense. A signed graph is* all-positive* (resp.,* all negative*) if all its edges are positive (negative); further, it is said to be* homogeneous* if it is either all-positive or all negative and* heterogeneous* otherwise. The* positive (negative) degree* of a vertex *v* ∈ *S* denoted by *d*^+^(*v*)(*d*^−^(*v*)) is the number of positive (negative) edges incident on the vertex *v* and *d*(*v*) = *d*^+^(*v*) + *d*^−^(*v*). The* negation* of a signed graph *η*(*S*) is obtained by reversing the sign of edges of *S*. Let *v* be an arbitrary vertex of a graph *S*. We denote the set consisting of all the vertices of Σ adjacent to *v* by *N*(*v*). This set is called the *neighborhood*  *set* of *v* and sometimes we call it as *neighborhood* of *v*. A *marked*  *signed*  *graph* is an ordered pair *S*^*μ*^ = (*S*, *μ*) where *S* = (Σ, *σ*) is a signed graph and *μ* : *V*(Σ)→{+, −} is a function from the vertex set *V*(Σ) of Σ into the set {+, −}, called a marking of *S*. *ℳ*_*S*_ denotes the set of all markings on vertices of *S*. For any vertex *v* ∈ *S*, *μ*_1_(*v*) = ∏_*u*∈*N*(*v*)_*σ*(*uv*) is called* canonical marking*. The marking on the vertices will be specified in the whole text as the case may be.

*N*_*∗*_(*t*) = {*v*_1_^*μ*^ ∈ (*V*(*S*^*μ*^)) : *tv*  is  an  edge  with  sign  *μ*}, *N*_*∗*_^+^(*t*) = {*v*^+^ ∈ (*V*(*S*^*μ*^)) : *tv*  is  an  edge}, and *N*_*∗*_^−^(*t*) = {*v*^−^ ∈ (*V*(*S*^*μ*^)) : *tv*  is  an  edge}. A vertex with a marking *μ* is denoted by *v*^*μ*^. A* cycle* in a signed graph *S* is said to be* positive* if the product of the signs of its edges is positive or, equivalently, if the number of negative edges in it is even. A cycle which is not positive is said to be* negative*.

A signed graph is* line balanced* or* balanced* if all its cycles are positive. The partition criterion to characterize the balance property of a signed graph is given by Harary. A marked graph is* vertex or point balanced* if it does not contain odd number of negative vertices. A signed graph *S* is* sign-compatible* [35] if there exists a marking *μ* of its vertices such that the end vertices of every negative edge receive “−” marks in *μ* and no positive edge in *S* has both of its ends assigned “−” mark in *μ*; it is* sign-incompatible* otherwise. A canonically marked graph *S* is said to be* canonically sign-compatible* (or* C-sign-compatible*) if end vertices of every negative edge receive “−” sign and no positive edge has both of its ends assigned “−” under *μ*.

The idea of* switching a signed graph* was introduced by Abelson and Rosenberg [36] in connection with structural analysis of social behaviour and may be formally stated as follows: given a marking *μ* of a signed graph *S*,* switching S* with respect to *μ* is the operation of changing the sign of every edge of *S* to its opposite whenever its end vertices are of opposite signs in *S*_*μ*_ (*also see* Gill and Patwardhan [37, 38]). The signed graph obtained in this way is denoted by (*S*)_*μ*_ and is called the *μ*-*switched signed graph* or just* switched signed graph* when the marking is clear from the context. Further, a signed graph *S*_1_* switches to signed graph S*_2_ (or that they are switching equivalent to each other), written as *S*_1_ ~ *S*_2_, whenever there exists *μ* ∈ *ℳ*_*S*_1__ such that (*S*_1_)_*μ*_≅*S*_2_, where “≅” denotes the isomorphism between any two signed graphs in the standard sense. Two signed graphs *S*_1_ and *S*_2_ are* cycle isomorphic* if there exists an isomorphism *f* : Σ_1_ → Σ_2_, where Σ_1_ and Σ_2_ are underlying graph of *S*_1_ and *S*_2_, respectively, such that the sign of every cycle *Z* in *S*_1_ equals the sign of *f*(*Z*) in *S*_2_.

**Figure 1:**
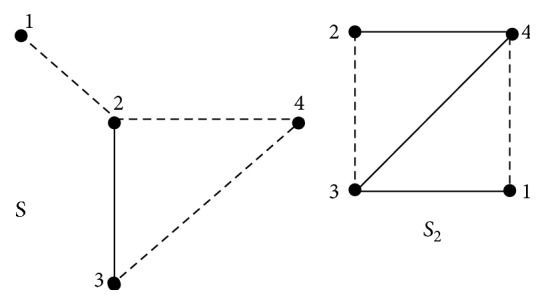
A signed graph and its 2-path signed graph.

**Figure 2:**
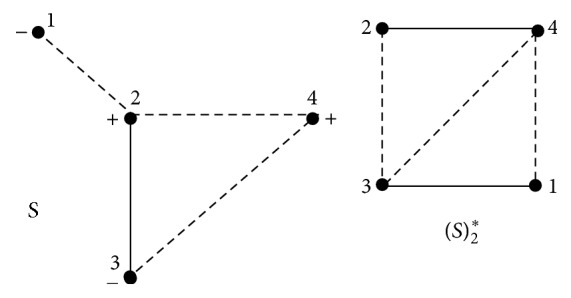
A signed graph and its 2-path product signed graph.

Assume that *S* = (*V*, *E*, *σ*) is a signed graph. We associate with *S* the* 2-path signed graph* [39] *S*#*S* = (*V*, *E*′, *σ*′) defined as follows: the vertex set is same as the original signed graph *S* and two vertices *u*, *v* ∈ *V*(*S*#*S*), are adjacent if and only if there exists a path of length two in *S*. The edge *uv* ∈ *V*(*S*#*S*) is negative if and only if all the edges in all the two paths in *S* are negative otherwise the edge is positive (see Figure 1). The* 2-path product signed graph *$S\hat{\#}S=(V,E^{\prime },\sigma^{\prime })$ [40] is defined as follows: The vertex set is same as the original signed graph *S* and two vertices $u,v\in V(S\hat{\#}S)$, are adjacent if and only if there exists a path of length two in *S*. The sign *σ*′(*uv*) = *μ*_1_(*u*)*μ*_1_(*v*), *μ*_1_ is canonical marking (see Figure 2).

**Property 1 (see [39]).**

A 2-subset {*v*_*i*_, *v*_*j*_} in a neighborhood of a vertex in a given signed graph *S* has property **P** if {*v*_*i*_^−^, *v*_*j*_^−^} ⊂ *N*_*∗*_(*v*_*k*_) for some *i*, *j*, *k* and for each *N*(*t*) containing *v*_*i*_, *v*_*j*_, {*v*_*i*_^−^, *v*_*j*_^−^} ⊂ *N*_*∗*_(*t*).

In the first section, we give a characterization of 2-path product signed graph, followed by a theorem of finding the degree of each vertex in $S\hat{\#}S$. Also, we find when a 2-path product graph is isomorphic and switching equivalent to its negation. Next, we find when $S\hat{\#}S$ is all negative for a given *S*. The following two sections are dedicated to signed graph properties sign-compatibility and canonical-sign-compatibility. The last section deals with the isomorphism and switching equivalence of the two types of 2-path graphs of signed graphs.

## 2. Characterization of 2-Path Product Signed Graph

We require the following theorems for the characterization of 2-path product signed graph.

**Theorem 2 (see [41]).**

A signed graph *S* is vertex balanced if and only if it is possible to assign signs to the edges of *S* such that the mark of any vertex *u* is equal to the product of the signs of the edges incident to *u*.

The following characterization of 2-path graphs was given by Acharya and Vartak.

**Theorem 3 (see [42]).**

A connected graph Σ with vertices *v*_*i*_, *i* = 1,…, *n* is of the 2-path graph form Σ = *H*#*H*, with some graph *H* if and only if Σ contains a collection of complete subgraphs Σ_1_, Σ_2_,…, Σ_*n*_ such that for each *i*, *j* = 1,…, *n*

- *v*_*i*_ ∉ Σ_*i*_;
- *v*_*i*_ ∈ Σ_*j*_⇔*v*_*j*_ ∈ Σ_*i*_;
- *v*_*i*_*v*_*j*_ ∈ Σ and there exists Σ_*k*_ containing *v*_*i*_*v*_*j*_.

**Theorem 4 (see [39]).**

A connected sigraph *S* with vertices *v*_*i*_, *i* = 1,…, *n* is a 2-path sigraph of some sigraph *S*′ if and only if *S* contains a collection of complete subsigraphs *S*_1_, *S*_2_,…, *S*_*n*_ with marked vertices *v*_*i*_^*μ*^, *μ* ∈ {+, −} such that, for each *i*, *j* = 1,…, *n*, the following hold:

- *v*_*i*_^*μ*^ ∉ *S*_*i*_;
- *v*_*i*_^*μ*_1_^ ∈ *S*_*j*_⇔*v*_*j*_^*μ*_2_^ ∈ *S*_*i*_, *i* ≠ *j*, *μ*_1_ = *μ*_2_;
- *v*_*i*_*v*_*j*_ ∈ *E*(*S*) with sign *σ*; then there exists *S*_*k*_ containing *v*_*i*_^*μ*_*i*_^, *v*_*j*_^*μ*_*j*_^ where *μ*_*i*_, *μ*_*j*_ ∈ {+, −} and if *σ*(*v*_*i*_, *v*_*j*_) = − then {*v*_*i*_, *v*_*j*_} is a **P** pair in *S*_*k*_.

The following proposition is evident from [43, 44].

**Proposition 5 .**

2-path product signed graph of a signed graph *S* is always balanced.

We give a characterization for 2-path product signed graph.

**Theorem 6 .**

A connected signed graph *S* with vertices *v*_*i*_, *i* = 1,…, *n* is of the 2-path product signed graph form $S=S^{\prime }\hat{\#}S^{\prime }$ with some signed graph *S*′ if and only if the underlying graph Σ is a 2-path graph and *S* is both line balanced and vertex balanced.

**Proof**

*Necessity*. Suppose *S* is of the 2-path product signed graph form $S=S^{\prime }\hat{\#}S^{\prime }$ with vertices *v*_1_, *v*_2_,…, *v*_*n*_. Now from Theorem 3, there exist *n* complete subsigned graphs such that (i), (ii), and (iii) hold. Let us consider the set *N*(*v*) of neighborhood of a vertex *v* in *S*′. For each vertex *v* in *S*′ there is a neighborhood *N*(*v*), hence *n* such subsets of neighborhoods. Clearly since we consider open neighborhood, *v* ∉ *N*(*v*), also if a vertex *u* ∈ *N*(*v*), then *uv* is an edge in *S* and hence *v* ∈ *N*(*u*). And if *uv* is an edge in *S* then *u* and *v* are adjacent to a vertex *w* in *S*′. That is *u*, *v* ∈ *N*(*w*) such that *σ*(*uv*) = *μ*_1_(*u*)*μ*_1_(*v*) since each vertex has a marking in *S*′. We know that *S*′ is a canonically marked signed graph; thus each vertex has a marking *μ*_1_. Now let *N*_*∗*_(*v*_*i*_) be the neighborhood of a vertex *v*_*i*_ with marked vertices retaining the marking from *S*′. Then clearly since all three properties (i), (ii), and (iii) of Theorem 3 are satisfied and also by Theorem 2, and Proposition 5, *S* is line balanced and vertex balanced.

*Sufficiency*. Let *S* be a given signed graph such that its underlying graph Σ is a 2-path graph and *S* is both line balanced and vertex balanced. Then by Theorem 3, it can be written as the union of *n* complete subsigned graphs *S*_1_, *S*_2_,…, *S*_*n*_ of marked vertices such that for each *i*, *j* = 1,…, *n*, (i), (ii), and (iii) hold. Now associate a vertex *v*_*i*_ ∉ *S*_*i*_ to *S*_*i*_ and join *v*_*i*_ to all the vertices in *S*_*i*_, *i* = 1,…, *n* and giving the edge *v*_*i*_*v*_*j*_ sign as that of the product of marking on *v*_*i*_ and *v*_*j*_ where *v*_*j*_ ∈ *S*_*i*_. Let the signed graph thus obtained be *S*′. Next we show that $S^{\prime }\hat{\#}S^{\prime }≅S$. Obviously $\Sigma^{\prime }\hat{\#}\Sigma^{\prime }≅\Sigma$, where Σ′ and Σ are underlying graph of *S*′ and *S*, respectively. Let *v*_*i*_*v*_*j*_ be an edge *S* with the sign *σ*; then *σ* = *μ*_1_(*v*_*i*_)*μ*_1_(*v*_*j*_), where *μ*_1_(*v*_*i*_) and *μ*_1_(*v*_*j*_) are markings on *v*_*i*_ and *v*_*j*_, respectively. By hypothesis, *v*_*i*_*v*_*j*_ ∈ *S*_*k*_ for some *k*. Hence we will associate a vertex *v*_*k*_ to *S*_*k*_ and let its marking be *μ*_1_. By definition, the sign of edge *v*_*i*_*v*_*j*_ in $S^{\prime }\hat{\#}S^{\prime }$ is *σ*′, *σ*′ = *μ*_1_(*v*_*i*_)*μ*_1_*μ*_1_(*v*_*j*_)*μ*_1_. That is *σ*′ = *σ* = *μ*_1_(*v*_*i*_)*μ*_1_(*v*_*j*_). Therefore, *S*′ is the signed graph such that $S^{\prime }\hat{\#}S^{\prime }≅S$.

The characterization of 2-path signed graph in Theorem 4 provides us with a mechanism to check if a given signed graph is 2-path of some signed graph, which is discussed in Algorithm 1. This has been rigorously studied elsewhere in the author's contribution which is fully devoted to 2-path signed graphs and its properties. Thus Algorithm 2 using Algorithm 1 detects if the given signed graph is 2-path product signed graph and find the original signed graph. In Algorithm 2, we use the adjacency matrix *A* = {*a*[*i*][*j*] : *i*, *j* ≤ *n*} and its order *n* to find the original signed graph.  Algorithm 3 is used to find the 2-path product signed graph for a given signed graph.

**Theorem 7 .**

If *u*^*μ*_1_^ ∈ *V*(*S*_*μ*_1__), *μ*_1_ ∈ {+, −} being the canonical marking of a vertex *u*, then the degree of the vertex *u* in $S\hat{\#}S$, for a given signed graph *S*, is given by the following:

- If *μ*_1_ = + then positive degree of *u* in $S\hat{\#}S=\left|{⋃}_{u^{+}\in N_{*}\left(x\right)}\left(N_{*}^{+}\left(x\right)-\left\{u\right\}\right)\right|$ and the negative degree of *u* = |⋃_*u*^+^∈*N*_*∗*_(*x*)_(*N*_*∗*_^−^(*x*)−{*u*})|.
- If *μ*_1_ = − then positive degree of *u* in $S\hat{\#}S=\left|{⋃}_{u^{-}\in N_{*}\left(x\right)}\left(N_{*}^{-}\left(x\right)-\left\{u\right\}\right)\right|$ and the negative degree is |⋃_*u*^−^∈*N*_*∗*_(*x*)_(*N*_*∗*_^+^(*x*) − {*u*})|.

**Proof**

By Theorem 6 the neighborhoods of a vertex of *S* gives the edges in $S\hat{\#}S$. That is, if *u*, *v* ∈ *N*_*∗*_(*x*) for some *x* ∈ *V*(*S*), then *uv* is an edge in $S\hat{\#}S$. Thus ⋃_*u*∈*N*_*∗*_(*x*)_  (*N*_*∗*_(*x*)−{*u*}) gives the number of vertices which form an edge with *u* in $S\hat{\#}S$. And since the marking is canonical in *S* thus positive edges in $S\hat{\#}S$ are given by vertices with same marking. Thus a vertex *u*^*μ*_1_^, *μ*_1_ ∈ {+, −} in $V(S\hat{\#}S)$ is given by the following:

- If *μ*_1_ = + then positive degree of *u* in $S\hat{\#}S=\left|{⋃}_{u^{+}\in N\left(x\right)}\left(N_{*}^{+}\left(x\right)-\left\{u\right\}\right)\right|$ and the negative degree of *u* = |⋃_*u*^+^∈*N*_*∗*_(*x*)_(*N*_*∗*_^−^(*x*) − {*u*})|.
- If *μ*_1_ = − then positive degree of *u* in $S\hat{\#}S=\left|{⋃}_{u^{-}\in N_{*}\left(x\right)}\left(N_{*}^{-}\left(x\right)-\left\{u\right\}\right)\right|$ and the negative degree is |⋃_*u*^−^∈*N*_*∗*_(*x*)_(*N*_*∗*_^+^(*x*) − {*u*})|.

**Theorem 8 .**

$S\hat{\#}S≅\eta (S)\hat{\#}\eta (S)$, if and only if *S* is a signed graph with each vertex of even degree.

**Proof**

  

*Necessity*. Let $S\hat{\#}S≅\eta (S)\hat{\#}\eta (S)$; then clearly the underlying graph Σ of *S* is such that $\Sigma \hat{\#}\Sigma ≅\eta (\Sigma )\hat{\#}\eta (\Sigma )$. Also since *S* is a canonically marked signed graph with each vertex of even degree, the mark on every vertex will be the product of edges incident to it. Let if possible *v* be a vertex with *x* number of positive edges incident to *v* and *y* be the number of negative edges incident to it. Then one of the following cases arises.

*Case 1*. Let *x* be even; then *y* is also even since the total number of edges incident to *v* is even. In negation of *S*, *y* will again be even (since *x* is even in *S*). Thus both retain the same marking for *v*.

*Case 2*. Let *x* be odd then *y* is odd. Clearly *μ*_1_(*v*) = −; also *μ*_1_(*v*) in *η*(*S*) is again negative. Thus in both $S\hat{\#}S$ and $\eta (S)\hat{\#}\eta (S)$ the marking of *v* is −.

Clearly, since marking on each vertex remains the same so their 2-path product signed graphs remain isomorphic.

*Sufficiency*. Let $S\hat{\#}S≅\eta (S)\hat{\#}\eta (S)$. Let if possible *v* be a vertex with odd degree. Let *x* be the number of positive edges incident to *v* and *y* be the negative edges incident to *v*; then the following cases arise:

- If *x* is odd then *y* is even. Consequently, *v* receives a positive marking in *S*, but in its negation the number of negative edges becomes odd and hence the sign is reversed.
- If *x* is even then *y* is odd. The marking in *S* and *η*(*S*) is again reversed.

Thus if the signed graph has odd degree vertices then the 2-path product graphs of *S* and *η*(*S*) are not isomorphic, which is a contradiction.

**Corollary 9 .**

For any signed graph *S*, $S\hat{\#}S\sim \eta (S)\hat{\#}\eta (S)$.

**Proof**

Clearly, $\Sigma \hat{\#}\Sigma ≅\eta (\Sigma )\hat{\#}\eta (\Sigma )$, where Σ is underlying graph of *S*. Next we know that $S\hat{\#}S$ is always balanced, for every signed graph *S*. Thus all cycles are positive and have even number of negative edges. Thus both $S\hat{\#}S$ and $\eta (S)\hat{\#}\eta (S)$ will possess cycles with even number of negative edges. Thus $S\hat{\#}S\sim \eta (S)\hat{\#}\eta (S)$.

**Theorem 10 .**

A 2-path product signed graph $S\hat{\#}S$ of a given signed graph *S* is all negative if and only if *S* is either a cycle of length 4*m* or a signed path and *S* does not contain a subsigned path *u*^+^, *w*^*μ*_1_^, *v*^+^ or *u*^−^, *w*^*μ*_1_^, *v*^−^, in *S* where *μ*_1_ ∈ {+, −}.

**Proof**

  

*Necessity*. Let for a given *S* its 2-path product signed graph $S\hat{\#}S$ be all negative. Clearly, the signed graph *S* can be a tree or a cycle. Now if *S* is not a cycle or tree then $S\hat{\#}S$ will consist of cliques which can not be all negative since cliques always consist of a cycle of length three which can never be all negative as 2-path product signed graphs are always balanced. Clearly, 2-path graph of a cycle of odd length is self-isomorphic. Thus the cycle of odd length can not generate all negative 2-path product graphs. The 2-path graphs of cycles of even length say 2*m* are disjoint cycles of length *m* each. So if *m* is odd then also the 2-path product signed graph can never be all negative. Thus, a cycle of length 4*m* can generate all negative 2-path product signed graphs. To produce all negative 2-path product signed graph $S\hat{\#}S$, *S* can not have subsigned path *u*^+^, *w*^*μ*_1_^, *v*^+^ or *u*^−^, *w*^*μ*_1_^, *v*^−^, on any subsigned path since then *uv* will be a positive edge in $S\hat{\#}S$.

Also if there is a tree *S* with a vertex of degree greater than two, then clearly it gives rise to a clique containing cycles of length three in $S\hat{\#}S$, thus having at least one positive edge. Hence the tree can not have a vertex of degree greater than two. Thus, it is a signed path.

*Sufficiency*. let *S* is either a cycle of length 4*m* or a signed path and *S* does not contain a subsigned path *u*^+^, *w*^*μ*_1_^, *v*^+^ or *u*^−^, *w*^*μ*_1_^, *v*^−^, where *μ*_1_ ∈ {+, −}. Clearly $S\hat{\#}S$ will be disjoint cycles in case of cycle except for *m* = 1 where it will be two disjoint signed paths. And in case of signed path $S\hat{\#}S$ will be disjoint paths. And since always for any subsigned path *u*, *w*, *v* in *S*, *u*, and *v* will occupy opposite mark in $S\hat{\#}S$, thus it makes edge *uv* negative in $S\hat{\#}S$. Thus $S\hat{\#}S$ is all negative.

## 3. Sign-Compatibility of 2-Path Product Signed Graphs

In this section, we give a characterization of sign-compatibility for 2-path product signed graphs.

**Theorem 11 (see [35]).**

**Figure 3:**
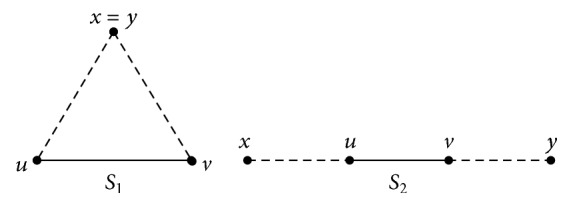
Acharya and Sinha two forbidden subsigned graphs for a sign-compatible signed graph.

A signed graph *S* is sign-compatible if and only if *S* does not contain a subsigned graph isomorphic to either of the two signed graphs in Figure 3, *S*_1_ formed by taking the path *P*_4_ : *x*, *u*, *v*, *y* with both the edges *xu* and *vy* negative and the edge *uv* positive, and *S*_2_ formed by taking *S*_1_ and identifying the vertices *x* and *y*.

**Theorem 12 .**

A 2-path product signed graph $S\hat{\#}S$ of a signed graph *S* is sign-compatible if and only if

- *S* does not contain a heterogeneous canonically marked triangle or *K*_1,3_;
- *S* does not consist of the canonically marked subsigned path *P*_7_ : *u*^+^, *w*^*μ*_1_^, *v*^−^, *x*^*μ*_1_^, *y*^−^, *z*^*μ*_1_^, *t*^+^ or *η*(*P*_7_) : *u*^−^, *w*^*μ*_1_^, *v*^+^, *x*^*μ*_1_^, *y*^+^, *z*^*μ*_1_^, *t*^−^, where *μ*_1_ ∈ {+, −}.

**Proof**

  

*Necessity*. Let 2-path product signed graph $S\hat{\#}S$ of a signed graph *S* be sign-compatible. To prove (i) and (ii), let *S* consist of a heterogeneous marked triangle *u*, *v*, *w*, *u*; then there exist two vertices with same mark and one vertex with different mark. Clearly the 2-path product signed graph $S\hat{\#}S$ will contain triangle *u*, *v*, *w*, *u* with two negative edges and one positive edge. Thus $S\hat{\#}S$ will not be sign-compatible, which is a contradiction. Again if *S* contains a heterogeneous canonically marked *K*_1,3_ then $S\hat{\#}S$ will consist of a forbidden triangle *S*_1_ in Figure 3. Hence (i) holds. Let if possible *S* consist of the canonically marked subsigned path *P*_7_ : *u*^+^, *w*^*μ*_1_^, *v*^−^, *x*^*μ*_1_^, *y*^−^, *z*^*μ*_1_^, *t*^+^ or *η*(*P*_7_) : *u*^−^, *w*^*μ*_1_^, *v*^+^, *x*^*μ*_1_^, *y*^−^, *z*^*μ*_1_^, *t*^−^, where *μ*_1_ ∈ {+, −}. Then $S\hat{\#}S$ will contain a forbidden *S*_2_ in Figure 3; thus $S\hat{\#}S$ will not be sign-compatible which is a contradiction to our assumption. Hence (ii) holds.

*Sufficiency*. Let (i) and (ii) hold. To show $S\hat{\#}S$ is sign-compatible, let if possible $S\hat{\#}S$ not be sign-compatible. Then $S\hat{\#}S$ must consist of subsigned graph isomorphic to Figure 3, which is not possible as then either (i) or (ii) does not hold true. Hence $S\hat{\#}S$ is sign-compatible.

## 4. C-Sign-Compatibility of 2-Path Product Signed Graphs

This section gives the C-sign-compatibility of 2-path product signed graphs.

**Proposition 13 (see [45]).**

Every C-sign-compatible signed graph is sign-compatible.

**Theorem 14 (see [45]).**

A signed graph *S* = (Σ, *σ*), is C-sign-compatible if and only if the following holds for *S*:

- For every vertex *v* ∈ *V*(*S*) either *d*^−^(*v*) = 0 or *d*^−^(*v*) = 1  (mod 2) and
- For every positive edge *e*_*k*_ = *v*_*i*_*v*_*j*_ in *S* either *d*^−^(*v*_*i*_) = 0 or *d*^−^(*v*_*j*_) = 0.

**Theorem 15 .**

A 2-path product signed graph $S\hat{\#}S$ of a signed graph *S* is C-sign-compatible if and only if

- *S* is sign-compatible;
- *S* does not contain a subsigned path *A* = *u*^−^, *w*^*μ*_1_^, *v*^−^, of vertices *u*, *w*, *v* where *μ*_1_ ∈ {+, −};
- if there exist a subsigned path *u*^+^, *w*^*μ*_1_^, *v*^+^ of vertices *u*, *w*, *v* in *S*; then either *d*^−^(*u*) = 0 or *d*^−^(*v*) = 0, where *μ*_1_ ∈ {+, −};

**Proof**

  

*Necessity*. Let $S\hat{\#}S$ be C-sign-compatible then clearly it is sign-compatible by Proposition 13. Let us suppose *S* contains a subsigned graph *u*^−^, *w*^*μ*_1_^, *v*^−^; then clearly *uv* is a positive edge in $S\hat{\#}S$ such that *d*^−^(*u*) ≠ 0 and *d*^−^(*v*) ≠ 0, which is a contradiction to the fact that $S\hat{\#}S$ is C-sign-compatible. Hence *S* does not contain subsigned path *u*^−^, *w*^*μ*_1_^, *v*^−^.

Let there exist a subsigned path *u*^+^, *w*^*μ*_1_^, *v*^+^ on vertices *u*, *w*, *v* in *S*, such that *d*^−^(*u*) ≠ 0 and *d*^−^(*v*) ≠ 0. Then *uv* is a positive edge in $S\hat{\#}S$ with both the vertices having negative degrees which is a contradiction to Theorem 14. Thus (i), (ii), and (iii) hold.

*Sufficiency*. Let (i), (ii), and (iii) hold. Then clearly for each positive edge *uv* in $S\hat{\#}S$ either *d*^−^(*u*) = 0 or *d*^−^(*v*) = 0. Hence by Theorem 14, $S\hat{\#}S$ is C-sign-compatible.

## 5. Isomorphism and Switching Equivalence of *S*#*S* and $S\hat{\#}S$

In this section, we give the switching equivalent and isomorphism for the two definitions of 2-path signed graphs.

**Theorem 16 (see [46]).**

Given a graph *G*, any two signed graphs are switching equivalent if and only if they are cycle isomorphic.

**Theorem 17 (see [39]).**

For a signed graph *S* of order n, its 2-path signed graph *S*#*S* is balanced if and only if for all sequences of vertices *x*_1_, *x*_2_,…, *x*_*N*_, 1 ≤ *N* ≤ *n* in *S* such that *x*_1_, *x*_2_ ∈ *N*(*t*_1_); *x*_2_, *x*_3_ ∈ *N*(*t*_2_); …; *x*_1_, *x*_*N*_ ∈ *N*(*t*_*N*_) for some *t*_1_, *t*_2_,…, *t*_*N*_ ∈ *V*(*S*); then the pairs *x*_*i*_, *x*_*i*+1_ ∈ *N*(*t*_*i*_), 1 ≤ *i* ≤ *N* having property **P** are even in each sequence.

**Theorem 18 .**

The 2-path signed graph *S*#*S* and 2-path product graph $S\hat{\#}S$ are switching equivalent if and only if *S*#*S* is balanced.

**Proof**

  

*Necessity*. if *S*#*S* and $S\hat{\#}S$ are switching equivalent then they are cycle isomorphic and hence *S*#*S* is balanced.

*Sufficiency*. Clearly, $\Sigma \hat{\#}\Sigma ≅\Sigma \#\Sigma$. Next, we know that $S\hat{\#}S$ is always balanced. For balanced *S*#*S*, each cycle of $S\hat{\#}S$ and *S*#*S* will be positive which implies that $S\hat{\#}S$ and *S*#*S* will be cycle isomorphic. Thus, by Theorem 16, $S\hat{\#}S$ and *S*#*S* are switching equivalent.

**Theorem 19 .**

The 2-path signed graph *S*#*S* and 2-path product graph $S\hat{\#}S$ are isomorphic, if and only if there exists subsigned path *u*^+^, *w*^*μ*_1_^, *v*^−^ or *u*^−^, *w*^*μ*_1_^, *v*^+^, *μ*_1_ ∈ {+, −} in *S*; then {*u*, *v*}, satisfies **P** property.

**Proof**

  

*Necessity*. For a signed graph *S*, let its 2-path signed graph *S*#*S* and 2-path product graph $S\hat{\#}S$ be isomorphic; here if *uv* is a negative (positive) in *S*#*S* then it is negative in $S\hat{\#}S$. All the pair of vertices {*u*, *v*} are negative in *S*#*S* and have property** P**. If there exist subsigned path *u*^+^, *w*^*μ*_1_^, *v*^−^ and *u*^−^, *w*^*μ*_1_^, *v*^+^ where *μ*_1_ ∈ {+, −} in *S* then *uv* is a negative edge in $S\hat{\#}S$ and thus {*u*, *v*} satisfies property** P**.

*Sufficiency*. Let there exist subsigned path *u*^+^, *w*^*μ*_1_^, *v*^−^ and *u*^−^, *w*^*μ*_1_^, *v*^+^ in *S* then {*u*, *v*} has property** P**. To show 2-path signed graph *S*#*S* and 2-path product graph $S\hat{\#}S$ are isomorphic. Clearly $\Sigma \hat{\#}\Sigma ≅\Sigma \#\Sigma$, Σ being the underlying graph of *S*. Thus we need to show that the sign convention remains the same in *S*#*S* and $S\hat{\#}S$. This is true since the end vertices of every negative edge of $S\hat{\#}S$ have property **P** and hence *uv* is a negative edge in *S*#*S*. And thus 2-path signed graph *S*#*S* and 2-path product graph $S\hat{\#}S$ are isomorphic.

## 6. Conclusion

In this paper, we have worked on 2-path product signed graph of a given signed graph *S*. A 2-path product signed graph is the signed graph where the vertex set is same as the original signed graph *S* and two vertices $u,v\in V(S\hat{\#}S)$ are adjacent if and only if there exists a path of length two in *S*. The sign *σ*′(*uv*) = *μ*_1_(*u*)*μ*_1_(*v*), *μ*_1_ being canonical marking. We give its algorithmic characterization along with its properties like sign-compatibility and C-sign-compatibility. Also, we find the isomorphism of 2-path product signed graph and its negation. We next find isomorphism of 2-path signed graph and 2-path product signed graphs.

## Figures and Tables

**Algorithm 1:**
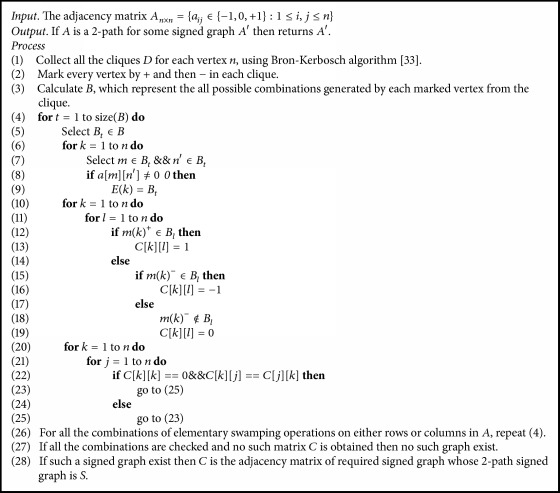
To check if the given signed graph is a 2-path of some other signed graph.

**Algorithm 2:**
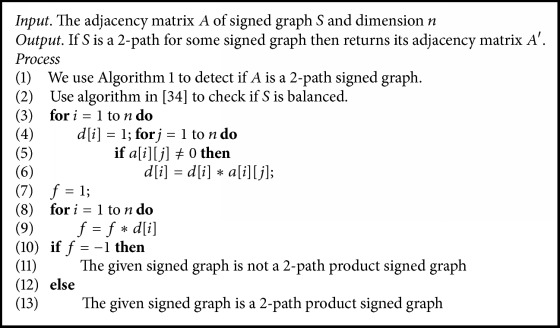
To check if the given signed graph is a 2-path product of some other signed graph.

**Algorithm 3:**
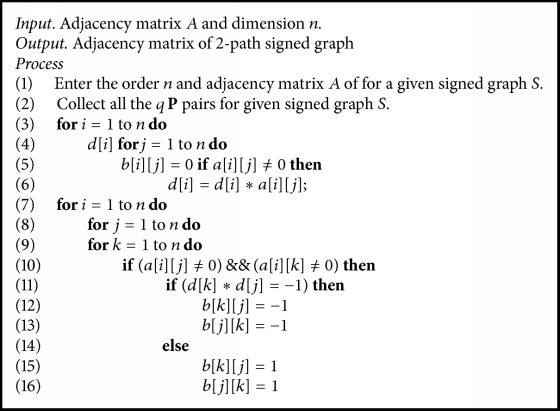
Algorithm to obtain a 2-path product signed graph for a given signed graph.
